# Transcription factors zeb1, twist and snai1 in breast carcinoma

**DOI:** 10.1186/1471-2407-11-73

**Published:** 2011-02-16

**Authors:** Ylermi Soini, Hanna Tuhkanen, Reijo Sironen, Ismo Virtanen, Vesa Kataja, Päivi Auvinen, Arto Mannermaa, Veli-Matti Kosma

**Affiliations:** 1 Department of Pathology and Forensic Medicine, Institute of Clinical Medicine, Pathology and Forensic Medicine, School of Medicine, University of Eastern Finland, Cancer Center of Eastern Finland, P.O. Box 1627, FI-70211 Kuopio, Finland; 2 Department of Clinical Pathology, Kuopio University Hospital, P.O. Box 1777, FI-70211 Kuopio, Finland; 3 Biocenter Kuopio, University of Eastern Finland, P.O. Box 1627, FI-70211 Kuopio, Finland; 4 Department of Oncology, Vaasa Central Hospital, 65130 Vaasa, Finland; 5 Department of Oncology, Kuopio University Hospital, P.O. Box 1777, FI-70211 Kuopio, Finland; 6 Institute of Biomedicine/Anatomy, University of Helsinki, P.O. Box 63, FI-00014 Helsinki, Finland

## Abstract

**Background:**

Epitheliomesenchymal transition (EMT) is the process where cancer cells attain fibroblastic features and are thus able to invade neighboring tissues. Transcriptional factors zeb1, snai1 and twist regulate EMT.

**Methods:**

We used immunohistochemistry to investigate the expression of zeb1, twist and snai1 in tumor and stromal compartments by in a large set of breast carcinomas. The results were compared with estrogen and progesterone receptor status, HER2 amplification, grade, histology, TNM status and survival of the patients.

**Results:**

Nuclear expression for twist was seen in the epithelial tumor cell compartment in 3.6% and for snai1 in 3.1% of the cases while zeb1 was not detected at all in these areas. In contrast, the tumor stromal compartment showed nuclear zeb1 and twist expression in 75% and 52.4% of the cases, respectively. Although rare, nuclear expression of twist in the epithelial tumor cell compartment was associated with a poor outcome of the patients (p = 0.054 log rank, p = 0.013, Breslow, p = 0.025 Tarone-Ware). Expression of snai1, or expression of zeb1 or twist in the stromal compartment did not have any prognostic significance. Furthermore, none of these factors associated with the size of the tumors, nor with the presence of axillary or distant metastases. Expression of zeb1 and twist in the stromal compartment was positively associated with a positive estrogen or progesterone receptor status of the tumors. Stromal zeb1 expression was significantly lower in ductal in situ carcinomas than in invasive carcinomas (p = 0.020). Medullary carcinomas (p = 0.017) and mucinous carcinomas (p = 0.009) had a lower stromal expression of zeb1 than ductal carcinomas. Stromal twist expression was also lower in mucinous (p = 0.017) than in ductal carcinomas.

**Conclusions:**

Expression of transcriptional factors zeb1 and twist mainly occur in the stromal compartment of breast carcinomas, possibly representing two populations of cells; EMT transformed neoplastic cells and stromal fibroblastic cells undergoing activation of zeb1 and twist due to growth factors produced by the tumor. However, epithelial expression of twist was associated with a poor prognosis, hinting at its importance in the spread of breast carcinoma.

## Background

Epitheliomesenchymal transition (EMT) is a process where epithelial cells attain fibroblastic properties. It has been postulated that in this way carcinoma cells are better able to invade to the surrounding structures and metastasize [1,2]. EMT is characterised by a downregulation of adhesion molecules, such as E-cadherin, and upregulation of genes typically found in myofibroblastic or fibroblastic cells such as α-smooth muscle actin or vimentin [1,2]. EMT is regulated by several transcription factors, such as snai1, slug, zeb1, twist, CarB-box-binding factor, Mesenchyme Forkhead 1 and Kruppel-like factor [2]. The expression of these transcription factors is modified and regulated by complex signaling networks present in the tumor microenvironment such as transforming growth factor β, notch or Wnt pathways [3]. There appears to be a hierarchy in the expression of these transcriptional factors, with snai1 being expressed at the onset of EMT whereas snai2, zeb1 and twist are induced later to maintain the migratory phenotype [3].

Zeb1 (Zinc-finger E-box-binding homeobox 1) is a transcriptional factor which contains two Kruppel-type zinc finger domains by which it becomes attached to target DNA sequences [4,5]. It induces EMT and has been shown to downregulate E-cadherin in epithelial cells [5]. Zeb1 also downregulates the polarity factor lethal giant larvae 2 (Lgl2) in colon cancer cell lines and promotes colon cancer cell metastasis [6]. It takes part in morphogenesis during the embryonic development by influencing the development of neural tissues, chondrocytes, skeletal muscle cells and hematolymphoid tissues [7]. Zeb1 is induced by transforming growth factor β1 and nuclear factor kappa beta (NF-kβ), it is unregulated by the hedgehog signalling pathway, its expression is influenced by hypoxia, but suppressed by the micro RNA miR-200 family [4,8,9]. Zeb1 expression is also induced by estrogen and progesterone [4]. In normal tissues, zeb1 mRNA expression is highest in bladder and uterus, but during embryonic development the highest mRNA levels are found in heart, lung and thymus [4].

Twist is a helix-loop-helix transcriptional factor which is known to promote EMT and downregulate E-cadherin [10]. It is important in head and limb development and a mutation of the twist gene causes the Saethre-Chotzen syndrome [11]. In hepatic carcinoma cell lines, twist induces cell motility and abrogates cellular adhesion and metastasis [10,12]. On the other hand, downregulation of twist by small interfering RNA (siRNA) in prostate carcinoma cells leads to a decreased metastatic potential and invasion of the tumor cells [13].

Snai1 is a transcriptional factor which downregulates the expression of E-cadherin, claudin 1 and cytokeratin 18 and upregulates vimentin and in this way it can contribute to EMT [14,15]. It is regulated by transcriptional factors such as NF-kappaB [16]. In mouse skin squamous cell carcinomas, its downregulation leads to retarded growth and invasiveness of the cancer cells [17]. Conversely, upregulation of snai1 expression is associated with a poor prognosis and metastatic potential in patients with ovarian or head and neck carcinomas [14,18]. In addition to being important in EMT and tumor invasion, snai1 plays a role in embryonic development and it can influence apoptosis, angiogenesis and matrix metalloproteinase 9 (MMP9) expression, i.e. factors which are important in promoting tumor growth [19,20].

Several cell line studies have shown that these transcriptional factors induce EMT and in this way can promote the metastatic potential of the malignant cells. In this study, we investigated the expression of zeb1, twist and snail in epithelial tumor and stromal compartments in a large prospective set of breast carcinomas to elucidate whether these factors are important in the spread of breast tumor cells *in vivo*. We used a tissue array material consisting originally of 556 breast carcinomas as the target material of which 511 were invasive and 45 of in situ type. The results were compared with the estrogen (ER) and progesterone receptor (PR) status, HER2 amplification, grade, histology, stage and survival of the patients.

## Methods

### Materials

The material consisted of 388 cases of invasive and in situ carcinomas. The details of the characteristics of the material are shown in Table 1. The material was collected from the files of the Kuopio University Hospital, Kuopio, Finland. All material had been fixed in 4% buffered formalin and embedded in paraffin. The slides were viewed and array blocks were constructed from representative areas of the tumors with at least one array sample representing the invasive front. The array blocks were constructed with a custom-built instrument (Beecher Instruments, Silver Spring, MD). The sample diameter of the tissue core in the array block was 1300 μm and three samples from tumor tissue of each case were studied. The diagnosis of the cases was based on the World Health Organization (WHO) classification of breast and female genital organs [21]. The presence of metastases was determined at the time of the operation. The collection of the material and the clinical features of the patients have been described in a previous study [22]. The research was approved by the ethical committee of Kuopio University and Kuopio University Hospital. According to Finnish legislation no informed consent to use paraffin embedded material in research is not needed from subjects if the material is large and retrospective, in such cases the consent can instead be applied from the National Supervisory Authority for Welfare and Health of Finland http://www.valvira.fi/; http://www.finlex.fi/fi/laki/ajantasa/2001/20010594. The consent to use the material was obtained from that institution.

**Table 1:** Data on the breast carcinoma cases studied

Histology	In situ	ductal invasive	lobular invasive	mucinous	medullary	others
	16	252	70	19	8	23
	Positive	Negative				
**ER**	279	87				
**PR**	230	150				
**Her2**	47	299				
	N0	N>0				
	217	165				
	T≤2	T>2				
	352	36				

### Immunohistochemistry for zeb1, twist and snai1

The immunostainings were performed as follows. Four-μm-thick tissue sections were cut from the paraffin-embedded blocks. After deparaffinisation and rehydration, the sections were heated in a microwave oven for 2 × 5 min in Trisaminomethane-Ethylenediaminetetraacetic Acid (Tris-EDTA) buffer (pH 9.0), incubated in a Tris-EDTA buffer for 20 min and washed twice for 5 min in phosphate buffered saline (PBS). Hydrogen peroxide (5%, 5 min) was used to block endogenous peroxidase. Non-specific binding was blocked with 1.5% normal serum in PBS for 35 min at room temperature. The sections were incubated overnight at 4°C with the mouse monoclonal anti - SnaiI, twist and zeb1 antibodies (dilutions 1:1000, 1:500 and 1:500, respectively). The twist antibody was purchased from Abcam (ab50887, Abcam, Cambridge, UK) and the mouse monoclonal zeb1 antibody from GenWay (clone 416A7H10, San Diego, CA, USA). The snai1 antibody has been characterised previously [23,24]. The slides were then incubated with a biotinylated secondary antibody and avidin-biotin-peroxidase complex (ABC Vectastain Elite Kit, Vector Laboratories, Burlingame, CA, USA). Careful rinses were performed with PBS at each step of the immunostaining procedure. The color was developed with diaminobenzidine tetrahydrochloride (DAP) (Sigma, St. Louis, MO, USA). The slides were counterstained with Mayer's haematoxylin, washed, dehydrated, cleared and mounted with Depex (BDH, Poole, UK). Ovarian tumor tissue with known positive Snail1, twist or zeb1 expression was used as a positive control. In the negative controls, the primary antibody was omitted.

The immunoreactivity for zeb1, twist and snail was separately analysed in the stromal fibroblastic and epithelial cell compartments of the tumor. Only nuclear positivity was considered significant. The immunoreactivity for fusiform stromal cells was semiquantified as follows;

0-5% = negative (0)

5-25% = weak positivity (1)

25-50% = moderate positivity (2)

50-75% = strong positivity (3)

75-100% = very strong positivity (4)

For epithelial tumor cells, only the presence of nuclear negativity (0) or positivity (1) was assessed.

The evaluation was performed by two pathologists (YS, RS) from three separate array samples. The median expression of zeb1 in nuclei of fusiform stromal cells was 1.6, and 1.3 for twist using the evaluation criteria presented above. For snai1, no stromal nuclear positivity was observed. These values were used as dividing points when separating the materials into two groups; low expression = 0 or high expression (1) (see Tables 2, 3, 4).

**Table 2:** Expression of zeb1, twist and snai1 in breast carcinoma

Stromal compartment	zeb1	Twist	Snail	Epithelial compartment	zeb1	Twist	Snail
High expression	188	135	0	Positive	0	14	12

Low expression	194	252	0	Negative	0	373	387

High expression > median value in estimations (see materials and methods)

Low expression ≤ median value in estimations (see materials and methods)

**Table 3:** Stromal expression of zeb1 and twist in relation to the estrogen receptor **status **in breast cancer

zeb1 stroma	ER+	ER-	Twist stroma	ER+	ER-
High expression	147	30		109	22

Low expression	128	56		169	65

	P = 0.003			P = 0.018	

High expression > median value in estimations (see materials and methods)

Low expression ≤ median value in estimations (see materials and methods)

**Table 4:** Stromal expression of zeb1 and twist in relation to the progesterone receptor in breast cancer

zeb1 stroma	PR+	PR-	Twist stroma	PR+	PR-
High expression	128	56		94	39

Low expression	100	92		135	111

	P < 0.001			P = 0.003	

High expression > median value in estimations (see materials and methods)

Low expression ≤ median value in estimations (see materials and methods)

### Statistical analysis

The statistical analyses were performed with SPSS for Windows software (SPSS, Chicago, IL, USA). Continuous data were compared using analysis of variance (ANOVA). When ANOVA results indicated that groups differed, post hoc comparisons were performed using two-tailed t-tests. Categorical data were compared using Fisher's exact test designed for small sample groups. Correlations were studied with Pearson´s correlation. Survival-data was analyzed using the Kaplan-Meier method with the use of the log-rank, Breslow and Tarone-Ware test. P-values less than 0.05 were considered statistically significant.

## Results

**Figure 1:**
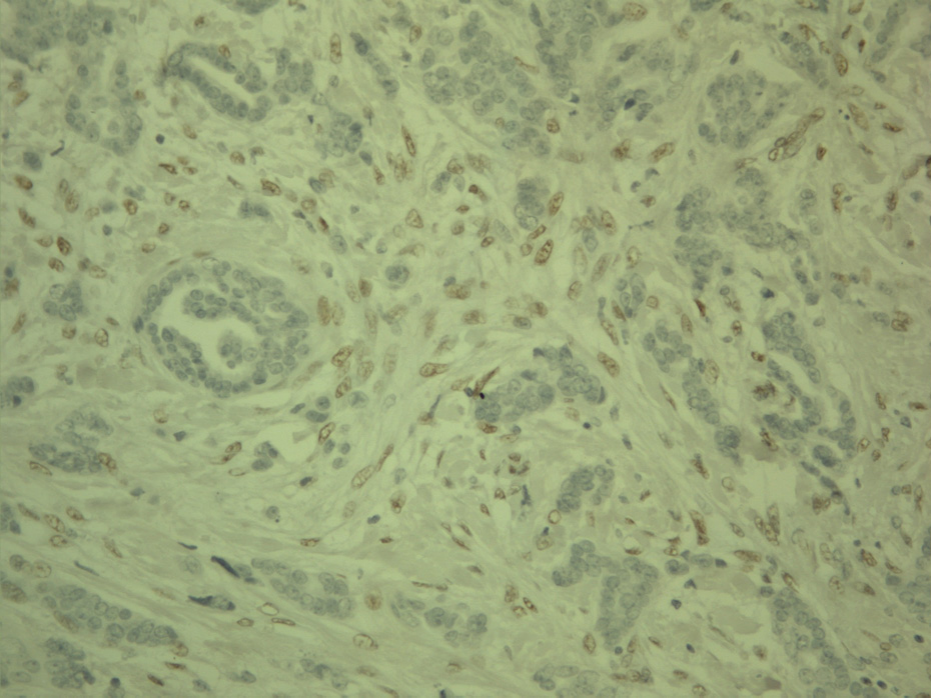
**Expression of zeb1 in breast carcinoma**. Strong nuclear expression is found in the stromal compartment while cells in the epithelial compartent are negative. The positive nuclei in stroma, however, show variation in size and display conspicuous nucleoli.

**Figure 2:**
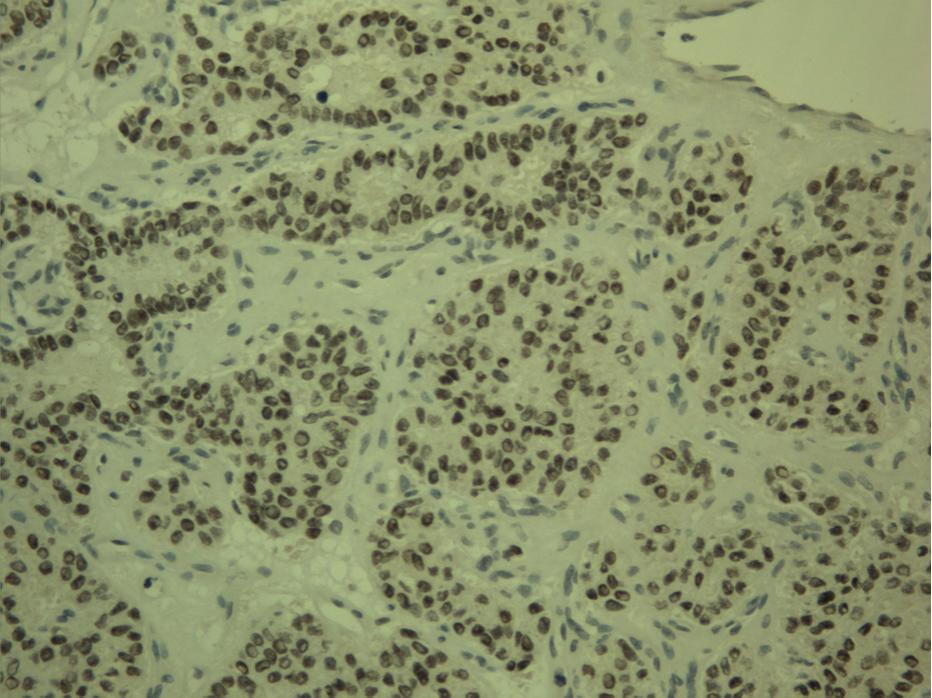
**Expression of twist in breast carcinoma**. In this case of a breast carcinoma, strong nuclear expression of twist is found in nuclei of the epithelial cell compartment of the cells.

**Figure 3:**
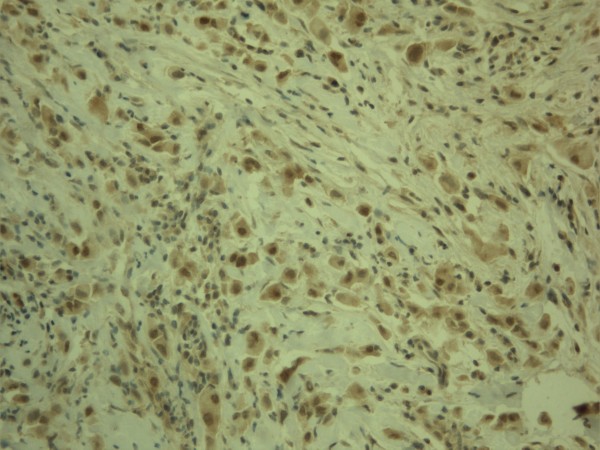
**Expression of snai1 in breast carcinoma**. Tumor cells show nuclear expression in the epithelial compartment while fusiform stromal cells are negative. There is some cytoplasmic positivity in both tumor and stromal cells.

**Figure 4:**
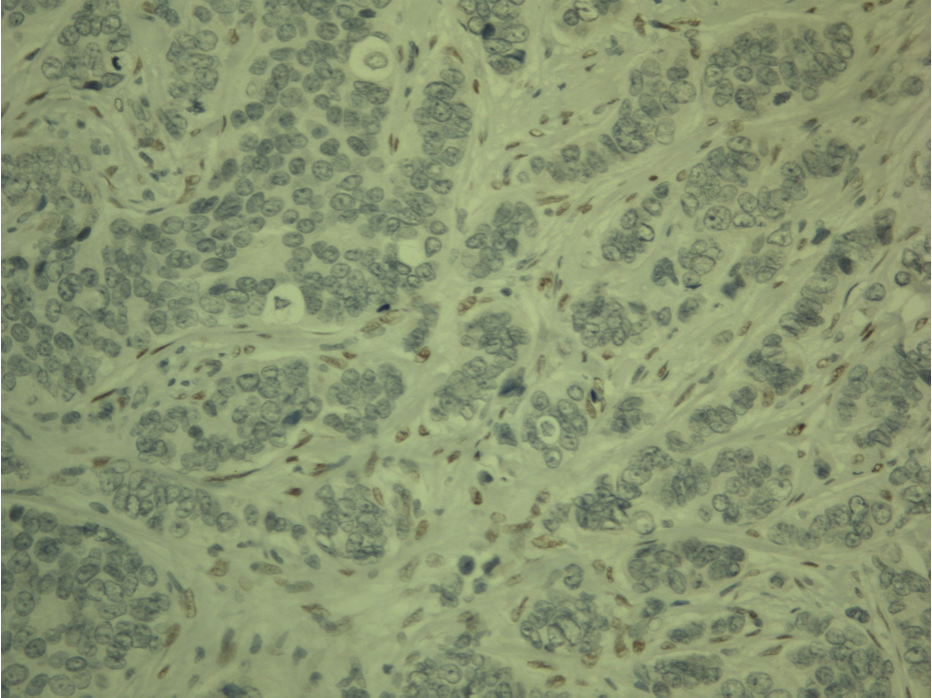
**Expression of twist in breast carcinoma**. In this case nuclear expression is found in the stromal compartment while epithelial tumor cells are negative.

No expression for zeb1 was found in breast cancer cells in the epithelial compartment (Figure 1). With twist and snai1, 14/387 (3.6%) and 12/387 (3.1%) showed nuclear positivity in epithelial cancer cells, respectively (Figure 2 and 3). In contrast, nuclear positivity in fusiform stromal cells was found in 260/347 (75.0%) for zeb1 and 187/357 (52.4%) for twist but with snai1, no evident nuclear positivity was detected in stromal fusiform cells (Table 1) (Figure 1 and 4). In the three separate array samples, the evaluations for zeb1, snai1 and twist correlated strongly positively (R = 532-613, p < 0.001 for all assessments). The interobserver variation for evaluation of stromal and nuclear expression was good (kappa = 0.548, p < 0.001).

### Expression in the stromal compartment

There was a significant positive association between zeb1 and twist expressions in stromal fusiform cells in breast carcinoma (p < 0.001) with stromal zeb1being significantly lower in ductal in situ carcinomas than in invasive carcinomas (p = 0.020). Medullary carcinomas (p = 0.017) and mucinous carcinomas (p = 0.009) had a lower stromal expression of zeb1 than ductal carcinomas. Stromal twist expression was also lower in mucinous carcinomas (p = 0.017) than in ductal carcinomas.

In the whole material, stromal zeb1 was associated with a positive estrogen (p = 0.003) and progesterone receptor status (p = 0.006) and inversely with HER2 amplification (p = 0.037) (Table 2 and 3). Stromal twist was also associated with a positive estrogen (p = 0.018) and progesterone receptor status (p = 0.009) (Table 2 and 3) but no association was found with HER2 (p = 0.14) Stromal zeb1 and twist did not associate with the presence of axillary metastases or the size of the tumors (p = 0.316 and p = 0.257)). There were more cases with weak stromal zeb1 positivity in grade I than in grade II tumors (p = 0.011). No such association was found for twist (p = 0.24). Interestingly, however, grade III tumors showed less stromal zeb1 positivity than grade II carcinomas (p = 0.001). No association was found between cytokeratin 5/6 and stromal zeb1 or twist expression (p = 0.923 and p = 0.952).

If HER2 positive cases were omitted from the analysis, then stromal zeb1 and twist positivities were still associated with a positive ER status (p = 0.010 and 0.015, respectively) and progesterone receptor status (p = 0.008 and 0.007, respectively). Stromal zeb1 was more commonly associated with grade I-II than grade III tumors (p = 0.042). Interestingly, no HER positivity in HER amplified cases was seen in the stromal cells suggesting that no positive evidence of EMT could be established in these cases.

### Expression in the epithelial compartment

Nuclear twist positivity in the epithelial carcinoma compartment was found in 7 ductal invasive, 4 lobular invasive, 1 tubular, 1 non-specified and one ductal carcinoma in situ. Most of the positive tumors were of grade II. No association was found with stromal twist or zeb1 positivity. There was no association with the estrogen receptor (p = 0.36) or HER2 status (p = 0.22). With the progesterone receptor, there was a near significant inverse association (p = 0.051). There was no association with the size of the tumor (p = 0.277) or axillary nodal status (p = 0.20).

Nuclear snai1 positivity in carcinoma cells was found in 7 ductal invasive, one lobular invasive, one mixed tubulolobular and one mucinous carcinoma. No significant association was found between snai1 and twist positivity (p = 0.638). Snai1 was inversely associated with a positive estrogen receptor status (p = 0.041), but not with the progesterone receptor (p = 0.58) nor with the size of the tumor (p = 0.301), presence of metastases (p = 0.57), grade of tumor (P = 0.10, grade I-II/III) or HER2 amplification (p = 0.215).

### Survival analysis

**Figure 5:**
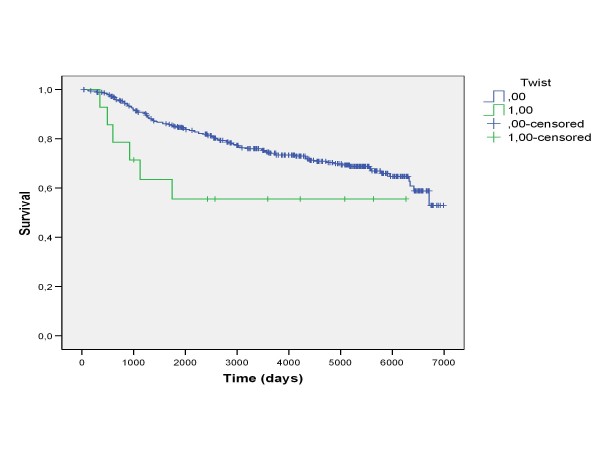
**Patients with tumors expressing nuclear twist positivity in the epithelial compartment had a poorer survival (p = 0.054 log rank, p = 0.013, Breslow, p = 0.025 Tarone-Ware)**.

In the whole material expression of zeb1 or twist in the stromal compartment did not associate with overall survival (p = 0.600 log rank, p = 0.668 log rank, respectively). Nuclear twist expression in the epithelial compartment was, however, associated with a poorer outcome of the patients (p = 0.054 log rank, p = 0.013, Breslow, p = 0.025 Tarone-Ware). (Figure 5) Nuclear snail expression in the epithelial compartment did not associate with survival (p = 0.96, log rank). In summary, nuclear snai1 and twist positivity had an association with survival in HER2 negative tumors (p = 0.065, log rank, p = 0.026, Breslow, p = 0.039 Tarone-Ware).

## Discussion

This study was undertaken to analyse the expression and role of transcription factors zeb1, twist and snai1 in human breast carcinoma. The study shows that nuclear expression of these transcription factors in breast carcinoma epithelial tumor cell compartment is uncommon with only 3.6% and 3.1% of cases being positive for twist and snai1 and with no positivity for zeb1. The cells in the stromal compartment, however, showed abundant nuclear positivity for zeb1 and twist, though no positive cases with snai1 were found where nuclear expression of fusiform stromal cells would have exceeded the 5% expression level considered as the limit for positive expression.

The low expression of zeb1, twist and snai1 in epithelial tumor cell compartment of breast carcinoma is surprising since these transcriptional factors are believed to be important in the spread of carcinomas through induction of EMT. Similar findings have been found in pancreatic adenocarcinomas where twist expression was lower in tumor cells than in non-neoplastic epithelial cells [25]. However, EMT is defined as a phenomenon where tumor cells lose their epithelial features, and thus a proportion of the cells in stromal tissue might represent transformed malignant cells. In fact, during EMT, epithelial cancer cells have been postulated to be transformed to mesenchymal type cells then invade the blood vessels or lymphatics after which they regain epithelial features at a metastatic site through mesenchymal-epithelial transformation [26]. Evidence for this kind of concept comes from the detection of similar genetic changes in both tumor and stromal cells in primary tumor sites while in metastases, stromal cells do not display these kinds of genetic changes [26]. Furthermore, epithelial cells have been shown to transdifferentiate to myofibroblastic cells in tissue fibrosis and cancer [27,28]. Our results could thus merely indicate that epithelial tumor cells initiate zeb1 and twist synthesis after being transformed to myoepithelial type cells. This is in line with the proposed hierarchy of the expression of such transcriptional factors in EMT [3,28] leading to that zeb1 and twist, which apparently maintain the migratory phenotype of tumor cells, are mostly detected in the stromal compartment in breast tumor tissue. On the other hand, evidently stromal fibroblast-derived cells are also activated to express zeb1 and twist which makes it difficult to differentiate these cell types from each other by morphologic means, a phenomenon which has also been described by others [28].

Even though the low frequency of cases with nuclear expression in epithelial type tumor cells, twist expression in these cells has clinical importance in breast carcinoma. Nuclear expression of twist was related to a poorer outcome of the patients. It has been shown previously that downregulation of twist in aggressive breast cancer cell lines leads to an abrogation of the invasive and metastatic phenotype of the cells and furthermore twist expression is associated with high grade breast tumors [3,29]. In addition to EMT twist has also other features which are linked to the growth or metastasis of tumor cells, i.e. it downregulates the expression of tissue inhibitors of metalloproteinase 1 (TIMP1) mRNA in Saos cells [30] promotes angiogenesis by inducing vascular endothelial growth factor (VEGF) and stimulates cancer cell migration [31,32]. Prevention of twist transcription in breast cancer blocked EMT, invasion and the development of multidrug resistance induced by adriamycin [33]. In line with this, expression of twist has been shown to be prognostically important also in other types of carcinomas such as cervix [34], hepatocellular [31], esophageal [35] and gastric carcinoma [36].

Nuclear expression of snai1 in the epithelial compartment was seen in 3.1% of cases which is considerably lower than has been reported in some other types of tumors. In endometrioid carcinomas, nuclear snail expression was found in 29% [14] and in ovarian tumors in 23-38% of cases [14,37]. Snai1 expression has variably been linked to prognosis in hormone-sensitive ovarian carcinomas [14,37]. Curiously, there are no extensive studies on clinical materials in breast cancer. Our present experiments reveal that in addition to being rare, nuclear snai1 expression in breast carcinoma does not seem to influence patient prognosis. Furthermore, it was not related to the size of the tumors or to the presence of metastases. The fact that snai1 was not observed in stromal cells might suggests that in contrast to some other tumors it does not play an important role in EMT of breast carcinoma. Clearer and stronger expression of snai1 has been detected in squamous cell carcinoma of the pharynx where stromal expression was associated with tumor size and prognosis (Jouppila-Mättö A, Tuhkanen H, Soini Y, Pukkila M, Närkiö-Mäkelä M, Sironen R, Virtanen V, Mannermaa A, Kosma V-M: Transcription factor Snail 1 expression and poor survival in pharyngeal squamous cell carcinoma, submitted). Evidently tumors of different sites and histology vary in their expression of snai1.

In our large set of breast carcinomas, no tumors were found with a positive nuclear expression of zeb1 in epithelial tumor cells. In contrast, zeb1 was found in fusiform stromal cells with a 75% proportion. In uterine tumors, ZEB 1 was expressed in stromal cells of low grade endometrial adenocarcinomas, but not in tumor cells [38]. In contrast, aggressive types of endometrial cancers also showed zeb1 expression in tumor cells [4,38]. Our results showed a lower expression of zeb1 in stromal cells in mucinous and medullary carcinomas than was the case in ductal carcinomas. These tumors have a slightly better prognosis than breast ductal or lobular carcinomas [21]. A similar association was observed for twist in mucinous carcinomas. The results could suggest that EMT activity would be lower in these histological types of tumors. Overall, stromal expression of zeb1 did not associate with the prognosis of the patients.

Stromal zeb1 expression was significantly lower in *in situ *type of breast carcinoma compared to invasive cases indicating that induction of zeb1 in stromal cells associates with the invasive phenotype. Some of these cells could represent transformed tumor cells undergoing EMT. Another proportion of the cell population could represent stromal fibroblastic cells undergoing activation of these transcription factors in response to the growth factors produced by the tumor tissue during the development of an invasive tumor. This kind of activation might stimulate stromal cells to transform to more motile alpha-smooth muscle actin-producing myofibroblasts. In fact, zeb1 serves as a transcriptional activator inducing genes such as alpha-smooth muscle actin, vimentin and collagens and heterozygous mutation of zeb1 gene leads to impaired smooth muscle actin and myosin expression [39].

In our set of breast carcinomas stromal expression of zeb 1 was associated with the estrogen and progesterone receptor status of the tumors. Previous studies have revealed that estrogen and progesterone may stimulate zeb1 expression. In ovarectomised mice, zeb1 was upregulated in uterine stroma and myometrium after progesterone or estrogen treatment [38]. Our results indicate that positive selection of the tumor cells for estrogen and progesterone receptors is reflected in the upregulation of zeb1 in stromal cells induced by the positive trophic stimuli of these hormones. Previously it has been shown that higher amounts of estradiol and estrone are present in estrogen positive tumors [40]. It remains to be clarified whether breast epithelial tumor cells per se do not express zeb1 even in hormone positive carcinomas. However, in experiments on ten breast and ovarian carcinoma cell lines, upregulation of the zeb1 gene was seen in only one cell line, suggesting that the zeb1 mRNA response in neoplastic cells has become deranged [4]. This is also reflected in findings on ovarian and uterine non-neoplastic and neoplastic tissues where zeb1 mRNA levels were associated with the estrogen level in non-neoplastic tissue but this association was lost in neoplastic tissues [4].

Curiously, however, stromal twist expression was not associated with a poorer survival of the patients nor was there any relation to tumor size or to the presence of metastases. Similarly no association was found for zeb1. The findings may be due to the fact that twist and zeb1 positive cells in the stroma represent a mixture of cell types, one proportion representing non-neoplastic activated fusiform fibroblastic cells, the other being EMT transformed tumor cells. Since these cells cannot be distinguished properly by morphology their relationships or even their relative numbers are difficult to measure. Thus the real quantity of EMT transformed cells in the stroma cannot be assessed by zeb1 or twist immunohistochemistry.

## Conclusions

Our results reveal that nuclear expression of zeb1, twist or snai1 is rare in epithelial tumor cell compartment of breast carcinoma. On the other hand, cells of the stromal compartment displayed abundant expression of zeb1 and twist but not snai1. Nuclear expression of twist in epithelial tumor cells was associated with a poorer prognosis of the patients indicative of its importance in the spread of breast carcinoma. However, stromal positivity of zeb1 or twist was not, however, associated with the size of the tumors, metastatic activity or prognosis of the patients. This may reflect the fact that the stromal compartment contains a mixture of zeb1 and twist positive cells, some representing EMT transformed neoplastic cells, the others being non-neoplastic activated fibroblasts.

## Competing interests

The authors declare that they have no competing interests.

## Authors' contributions

YS carried out and supervised the immunohistochemical stainings and was aided by IV, YS, AM, VK and PA collected the study material, YS, VMK and AM designed the study, YS and RS analysed the results and performed the statistical analyses, YS drafted the manuscript and was aided in this by VMK, IV and AM. All authors read and approved the final manuscript.

## Pre-publication history

The pre-publication history for this paper can be accessed here:

http://www.biomedcentral.com/1471-2407/11/73/prepub
